# A reappraisal of *Adinobotrys* Dunn (Fabaceae) with two new combinations

**DOI:** 10.3897/phytokeys.165.58477

**Published:** 2020-10-28

**Authors:** James A. Compton, Brian D. Schrire

**Affiliations:** 1 Spilsbury Farm, Tisbury, SP3 6RU, UK Spilsbury Farm Tisbury United Kingdom; 2 Comparative Plant and Fungal Biology Department, Royal Botanic Gardens, Kew, Richmond, Surrey, TW9 3AE, UK Royal Botanic Gardens London United Kingdom

**Keywords:** *
Adinobotrys
*, *
Callerya
*, Leguminosae, morphological key, Tribe Wisterieae

## Abstract

Two new species from Borneo that have been described in the genus *Callerya* are shown here to belong in *Adinobotrys*. The new combinations *A. katinganensis* and *A. sarawakensis* have consequently been made, bringing the total number of species in the genus to four. A morphological key and taxonomic conspectus is provided for all species.

## Introduction

Schot (1994: 15) included *Pongamia atropurpurea* Wall., in her revision of the genus *Callerya* Endl. (Endlicher 1843) as *Callerya atropurpurea* (Wall.) Schot., the species having already been segregated from *Millettia* (Wight & Walker-Arnott 1834) and *Padbruggea* Miq. (Miquel 1855) into the genus *Adinobotrys* Dunn, on the basis of apparently having a stipitate ovary and one-seeded pods (Dunn 1911: 194). Schot regarded *Adinobotrys* to be synonymous with *Padbruggea* Miq. The distinction between *Adinobotrys* and the genera *Padbruggea* and *Whitfordiodendron* Elmer, 1910 was fully discussed in Compton et al. (2019: 35). *Adinobotrys* was typified on the species *A. atropurpureus* (Wall.) Dunn by Geesink (1984: 83) based on *Pongamia atropurpurea* Wall. (Wallich 1830).

Research based on morphology, NrDNA and CpDNA sequence data has led to the redefinition of the *Callerya* Group of genera within the Inverted Repeat-lacking clade (IRLC) of legumes as an enlarged Tribe Wisterieae Zhu (Zhu 1994) comprising 36 species within 13 genera (Compton et al. 2019). The same research revealed that the genus *Adinobotrys* (Dunn 1911) was sister to *Glycyrrhiza* L., (Linnaeus 1753) and Tribe Wistereae and that another tree species *Callerya vasta* (Kosterm.) Schot, also belonged in *Adinobotrys* (Compton et al. 2019: 20).

*Adinobotrys* as circumscribed by Compton et al. (2019) comprised two species; *A. atropurpureus* (Wall.) Dunn and *A. vastus* (Kosterm.) J.Compton & Schrire. Adema (2019) has subsequently described two new tree species in *Calleryasens.lat.* (Schot 1994) from Borneo, which are placed here in *Adinobotrys*, bringing the total to four species.

Several morphological characters separate *Adinobotrys* from *Calleryasens.str.* (Compton et al. 2019: 35): *Adinobotrys* comprises species of evergreen trees (vs. lianes in all genera of Tribe Wisterieae); its bracteoles are persistent, placed on the calyx (vs. mostly caducous and placed on the pedicel in *Calleryasens.str.*); its standards are glabrous (vs. sericeous in *Calleryasens.str.*) and its wing petals are ± equal to the keel in length (vs. much shorter than the keel in *Calleryasens.str.*).

Although the position of *Adinobotrys* with respect to its sister genus *Glycyrrhiza* and the other genera in tribe Wisterieae is not yet fully resolved (see discussion in Compton et al. 2019), it is nevertheless fully supported in being excluded from Tribe Wisterieae and is morphologically distinct from *Calleryasens.str.* as noted above. Accordingly we make the two new combinations here in *Adinobotrys* with a new key emended from Adema (2019).

## Taxonomic conspectus

### Adinobotrys

Taxon classificationPlantaeFabalesFabaceae

Dunn, Bull. Misc. Inform. 1911: 194 (1911)

D9AFF004-3C5B-5659-B247-E7EF619942CE

≡ Millettia Sect. Nothomillettia Miq., Fl. Ned. Ind., Eerste Bijv. 2: 301 (1861).
≡ Millettia subgen. Nothomillettia (Miq.) Kurz, J. Asiat. Soc. Bengal, Pt. 2, Nat. Hist. 45(2): 273 (1876).

#### Key to species of *Adinobotrys*

**Table:** 

1	Leaves with 5–9 leaflets, lateral leaflets ± equal-sided at base; flowers 12–17 mm long; pod not inflated, flattened, narrowly elliptic to narrowly obovate; seeds 2–4, flattened lenticular, 3–9 mm thick (unknown for *A. vastus*)	**2**
–	Leaves with 7–11 leaflets; lateral leaflets oblique at base; flowers 17–20 mm long; pod inflated, elliptic to obovate, 7–20 × 3–6 cm; seeds 1–2, ovoid, 20–26 mm thick	***A. atropurpureus***
2	Indumentum grey; bracteoles at base of calyx	**3**
–	Indumentum brown; floral bracts 1.6 mm long; bracteoles halfway along calyx; pod 14–23 × 2.5–3 cm	***A. katinganensis***
3	Floral bracts 2.8–5 mm long; calyx sericeous; disk c. 2 mm high; pod 19–24 × 2.5 cm	***A. sarawakensis***
–	Floral bracts 1.2 mm long; calyx almost glabrous; disk c. 0.5 mm high; pod 23–24 × 4–4.5 cm	***A. vastus***

### Adinobotrys atropurpureus

Taxon classificationPlantaeFabalesFabaceae

(Wall.) Dunn, Bull. Misc. Inform. 1911(4): 194 (1911)

DF59AE45-5045-51D0-97D0-59C71AABAE29

≡ Pongamia atropurpurea Wall. Pl. As. Rar. 1(4): 70 t. 78 (1830). Type: Myanmar. “Martaban [Mottama] ad Amherst [Kyaikkami] 15 July 1827”, *Wallich* Cat. No. *5910*, holotype K! [K-000881026]; isotypes BM! [BM-000997335]; BO n.v.; CAL x 2 n.v.; P! [P-02141756]
≡ Millettia atropurpurea (Wall.) Benth. Pl. Jungh. [Miquel] 2: 249 (1852).
≡ Phaseoloides atropurpureum (Wall.) Kuntze, Revis. Gen. Pl. 1: 201 (1891).
≡ Whitfordiodendron atropurpureum (Wall.) Dunn, Bull. Misc. Inform. Kew 1912(8): 364 (1912).
≡ Callerya atropurpurea (Wall.) Schot, Blumea 39(1–2): 15 (1994).
= Millettia paniculata Miq., Fl. Ned. Ind., Eerste Bijv. 2: 301 (1861). Type: Indonesia, Sumatra “Sumatra orient. in prov. Palembang prope Kebur Lahat (T.)” 3675 H.B. Leguminosae, Masiboengan, Hortus Botanicus 023149 Utrecht, *J.E.Teijsmann s.n.*, holotype U! [U-0003669].
= Padbruggea pubescens Craib, Bull. Misc. Inform. Kew 1927(2): 61 (1927) Type: Thailand, Prov. Nakawn Panom [Nakhon Phanom], Ta Uten, elev. 1200 m, 15 February 1924, tree, fls pink. Ki Mo., *A.F.G.Kerr 8457*, holotype K! [K-000881016]; isotypes ABD n.v.; BM! [BM-000997332]; E! [E00275433].
≡ Whitfordiodendron pubescens (Craib) Burkill, Bull. Misc. Inform. Kew 1935(5): 319 (1935).
≡ Callerya atropurpurea (Wall.) Schot var. pubescens (Craib) P.K.Lôc, Bot. Zhurn. (Moscow & Leningrad) 81(10): 98 (1996).

#### Illustrations.

Lôc and Vidal in Fl. Cambodge, Laos & Vietnam 30: 34, t. 8 [9 – 11] (2001). https://singapore.biodiversity.online (in Home Page enter *Callerya atropurpurea*).

#### Distribution.

Cambodia; India; Indonesia (Java, Sumatra); Laos, Malaysia (Malay Peninsula); Myanmar; Thailand and Vietnam.

#### Habitat.

A component of evergreen forests from sea level to 1200 m.

### Adinobotrys katinganensis

Taxon classificationPlantaeFabalesFabaceae

(Adema) J.Compton & Schrire comb. nov.

30FD7605-6B02-5D95-8881-1C900DE880F9

urn:lsid:ipni.org:names:77212569-1

≡ Callerya katinganensis Adema, Blumea 64(3): 275 (2019). Type: Indonesia, Borneo, Kalimantan, Upper Katingan river, c. 96 km west of Batu Badingding, K. T. C. logging area west of base camp, elev. c. 200 m. 20 December 1982. *J.P.Mogea* 4276 holotype L! [L-0772470]; isotype L! [L-0772469]; BO n.v.

#### Note.

For a full description, details of habitat and ecology see Adema (2019: 275).

### Adinobotrys sarawakensis

Taxon classificationPlantaeFabalesFabaceae

(Adema) J.Compton & Schrire comb. nov.

78870C9D-579E-5A2C-A4F1-EA5BEDF7415A

urn:lsid:ipni.org:names:77212570-1

≡ Callerya sarawakensis Adema, Blumea 64(3): 276 (2019). Type: Malaysia, Borneo, Sarawak, third division, Bukit Raya, Kapit elev. 300 m. 26 November 1963. *P.P.K.Chai S-18911* holotype L! [L-0772465]; isotypes BO! [BO-1711732]; BO! [BO-1714048]; KEP! [KEP-195256]; MEL n.v.; SING! [SING-0263900]; SING! [SING-0263901].

#### Note.

For a full description, details of habitat and ecology see Adema (2019: 276).

### Adinobotrys vastus

Taxon classificationPlantaeFabalesFabaceae

(Kosterm.) J.Compton & Schrire, Phytokeys 125: 50 (2019)

27EDDFBE-7E3A-5E33-8F5A-572062E10FB3

≡ Millettia vasta Kosterm., Reinwardtia 5: 349 (1960). Type: Indonesia, Kalimantan [Borneo], Belajan River near Muara Lempong, June 1956, *A.J.G.H.Kostermans 12516A*, holotype BO! [BO-1249898]; isotypes BM! [BM-000997327]; K! [K-000880991]; L! [L-0018805]; P! [P-03081895]
≡ Callerya vasta (Kosterm.) Schot, Blumea 39(1–2): 36 (1994).

#### Distribution.

Borneo: Brunei; Indonesia (Kalimantan); Malaysia (Sabah, Sarawak).

#### Habitat.

Component tree in woods and forests from sea level to 250 m.

## Supplementary Material

XML Treatment for Adinobotrys

XML Treatment for Adinobotrys atropurpureus

XML Treatment for Adinobotrys katinganensis

XML Treatment for Adinobotrys sarawakensis

XML Treatment for Adinobotrys vastus
